# Latinos and Latinas in Communal Settings: A Grounded Theory of Recovery

**DOI:** 10.3390/ijerph6041317

**Published:** 2009-03-31

**Authors:** Josefina Alvarez, Leonard A. Jason, Margaret I. Davis, Bradley D. Olson, Joseph R. Ferrari

**Affiliations:** 1 Adler School of Professional Psychology, 65 E. Wacker Place, Suite 2100, Chicago, IL 60601, USA; 2 DePaul University, Center for Community Research, 990 Fullerton Avenue, Suite 3100, Chicago, Illinois 60614, USA; 3 Northwestern University, School of Education and Social Policy 2120 Campus Drive Evanston, IL 60208, USA; 4 DePaul University, Department of Psychology, 2219 N. Kenmore Ave., Chicago, Il. 60614, USA

**Keywords:** Grounded Theory, Recovery homes, Addiction, Latino/Latina

## Abstract

Semi-structured interviews were conducted with 12 Latino/a residents of a mutual help residential recovery program (Oxford House) in order to elicit their experiences of the program’s therapeutic elements. A model of recovery emerged from the analysis including several themes supported by existing literature: personal motivation and readiness to change, mutual help, sober environment, social support, and accountability. Consistent with a broad conceptualization of recovery, outcomes included abstinence, new life skills, and increased self-esteem/sense of purpose. Most participants were the only Latino/a in their Houses; however, cultural differences did not emerge as salient issues. The study’s findings highlight potential therapeutic aspects of mutual-help communal recovery programs and suggest that English-speaking, bicultural Latinos/as have positive experiences and may benefit from participating in these programs.

## 1. Introduction

Research consistently supports the importance of environmental factors such as support from family and abstinent peers, the ability to find employment, and the absence of relapse triggers as predictors of long-term outcomes for individuals in recovery [1,2]. Perhaps the most widely studied predictor of long-term abstinence from drug and alcohol use is 12-step affiliation. Numerous studies support a relationship between participation in 12-step groups such as Alcoholics Anonymous (AA) and Narcotics Anonymous (NA) and long-term abstinence [3–5]. Additionally, research has revealed some of the therapeutic mechanisms of 12-step participation, including support from abstinent peers as well as increased motivation, self-efficacy and coping skills [6].

Latinos/as are the largest ethnic group in the United States [7]; however, the factors that may affect their long-term outcomes following substance abuse treatment have not been adequately studied. Research indicates that many Latinos/as use 12-step groups and endorse these groups’ principles and practices [8]. There are Spanish speaking Alcoholics Anonymous groups throughout the U.S., and AA has become widespread in Latin America [9,10]. However, published studies of 12-step participation among Latinos/as, which have focused on AA, have yielded contradictory findings regarding meeting attendance and program involvement [8,11,12]. Additionally, few studies have explored the impact of 12-step participation on recovery among Latinos and Latinas [11].

Mixed findings on meeting attendance and 12-step participation may be explained by the heterogeneity of Latinos/as living in the U.S. It is possible that some Latinos and Latinas may not participate in or benefit from 12-step groups because of language and cultural differences from other 12-step participants [10]. Additionally, because Latinos/as are less likely than Caucasians to access treatment programs and tend to leave substance abuse treatment prematurely, many individuals from this group may not have an opportunity to become exposed to 12-step groups [13]. Finally, many Latinos and Latinas may rely on other sources of support, such as family members, close friends, and clergy [10]. Because the effectiveness of mutual help interventions such as 12-step programs has been demonstrated, more research is needed to understand predictors of utilization of and outcomes in these groups among Latinos and Latinas.

Oxford House is an innovative mutual help program that provides housing and 24 hour support to persons in recovery who live together in all-men or all-women homes. Professionals are not involved in the Houses and there are no limits on length of stay. There are over 1,300 Oxford Houses throughout the United States, Canada, and Australia [14]. Houses are run democratically based on 80% approval of House policies by the residents. Residents also vote on all decisions including whether or not to accept an applicant or evict a resident. Every six months, individuals living in the House elect a president, treasurer, and comptroller, who are responsible for conducting meetings, keeping track of finances, and paying bills. To avoid eviction, residents must abstain from using substances, pay rent, help with various chores, and avoid disruptive behaviors [14]. Oxford House guidelines encourage residents to participate in 12-step groups and to seek other types of treatment interventions [14]. Because Oxford House requires residents to abstain from substance use, individuals typically join this program after completing inpatient detoxification or residential treatment programs. Oxford House fits in along the continuum of care of services available to people from professional support to self-help programs. There are only 1,300 Oxford Houses in the US, so they are limited in availability for many people.

Research supports the effectiveness of Oxford House as an aftercare intervention for individuals attempting to recover from drug and alcohol abuse [15–18]. For example, a randomized study of Oxford House found that individuals who participated in this program after completing residential treatment were less likely to use substances and be incarcerated and more likely to be employed, when compared to individuals who received other community-based aftercare interventions [18]. While the therapeutic mechanisms of Oxford House are not well-understood, a national study of this program indicates that living in Oxford House results in increased abstinence self-efficacy which predicts abstinence [16]. Diverse groups of recovering individuals appear to benefit from living in Oxford House, including men and women, African Americans and European Americans, Deaf individuals, ex-offenders, and those experiencing co-occurring mental health problems [19–23].

Latinos and Latinas are under-represented in Oxford House [16] and there may be a number of explanations for their low rates of participation in this program. Alvarez, Jason, Davis, Ferrari, and Olson [24] reported that Latinos/as were generally not aware of the availability of Oxford House as a recovery alternative and were primarily referred by substance abuse counselors. In addition to the lack of exposure to Oxford House, other potential explanations for the under-representation of Latinos/as in Oxford House may include language and cultural differences and concerns about being in the minority [24]. However, a small sample of Latinos and Latinas found Oxford House helpful in their recovery and felt comfortable living in Houses where they were in the minority [24]. The current study explored residents’ experiences of the therapeutic components of their residence in Oxford House.

## 2. Methods

### 2.1. Procedures

This study collected qualitative interviews using grounded theory methodology, which is an excellent way to meet the purpose of the study as this approach develops a model based on the participants’ perspectives and experiences. Purposeful sampling [25] was employed to select participants for the current study. In order to sample a diverse group of Latino/a Oxford House residents, the goal of the sampling approach was to select men and women from various U.S. regions representing various age groups and places of birth. Participants were recruited via letters and telephone calls by the first author from a sample of 31 Latino/a individuals participating in a national study of Oxford House (see Jason, Davis, *et al*. [16] for a description of the study’s methodology; only 3.5% of this sample was Latino/a). These participants were then asked to refer other Latino/a Oxford House residents.

Participants were interviewed individually by the first author using a semi-structured interview guide [24]. Interviews were 60 minutes in duration, and were conducted either face-to-face (for the five participants located in the Midwest) or by telephone (for all other participants). Appendix A contains the interview guide. Interviews were tape-recorded and transcribed verbatim. Two individuals elected to be interviewed in Spanish and another participant began the interview in English and switched to Spanish toward the end of the hour. Spanish interviews were translated by a bilingual research assistant and back-translated by the first author prior to coding. Interview transcripts were analyzed using NVIVO [26].

### 2.2. Data Analysis

The first author analyzed the interview data using grounded theory methodology [27,28]. This approach allows for a conceptual analysis of the participants’ responses, with the goal of developing a model based on their perspectives and experiences [28,29]. Coding began with microanalysis [28], a line by line analysis of participants’ statements regarding therapeutic components of Oxford House. Then, both open (naming concepts based on participants’ responses) and axial coding (finding relationships among categories that emerge from the data to develop a theory) were utilized [28]. Because the researchers’ aim was to understand the meaning given by participants to their experiences, analysis followed a more constructivist grounded theory approach proposed by Charmaz [27]. In a sense, constructivist approaches place priority on the data collection and interpretation process as being influenced and created by the shared experiences of the researcher and the participant.

The third author independently coded the participants’ responses, using the grounded theory approach just described. Differences in coding were discussed until consensus was reached by the two coders. Findings were then presented to a larger research team that included the other authors. Finally the authors attempted to make the study’s findings available to participants for their review. However, only one participant responded and the others could not be located.

## 3. Results

Twelve Oxford House residents (nine men and three women) participated in the study. Three participants were 29 years old, seven were in their 30’s, and two were in their 40’s. Five individuals were recruited from the Midwest, three from the Southwest, three from the Northeast, and one from the Northwest. In terms of education, five participants had attended college but had not graduated, two were high school graduates, four had attended elementary school, and one did not reveal his educational background. Nine participants lived in Oxford House for less than one year, two lived in their House for one to two years, and one individual had spent three years in Oxford House.

Seven individuals reported using multiple substances, four reported using primarily one substance (three used mainly alcohol and one cocaine), and one participant did not reveal his substance use history. In addition, nine participants reported being involved in the criminal justice system at the time they were referred to Oxford House. All but one of the participants reported that they were abstaining from using substances at the time of the study. One participant, who had left the Oxford House, reported using marijuana and alcohol at lower rates than before joining the program.

In terms of ethnicity, six participants identified themselves as Puerto Rican, five as Mexican American, and one reported her ethnicity as Latin American and Asian American. Nine of the study’s participants were born in the United States; the other three were born in Puerto Rico and moved to the States as teenagers or young adults. Seven individuals reported that they spoke at least some Spanish, but all 12 participants reported that English was their primary language.

**Figure 1.**
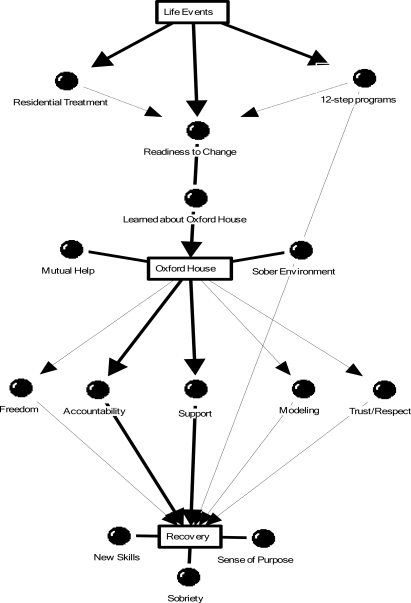
Therapeutic Components of Oxford House.

Several themes emerged from data analysis; they will be summarized below. Figure 1 presents a model of the therapeutic elements of Oxford House based on the study’s findings. As shown in Figure 1, the model consists of three parts: experiences that contributed to motivation for recovery prior to entering Oxford House, therapeutic aspects of the Oxford House program, and components of recovery.

### 3.1. Experiences Prior to Entering Oxford House

#### 3.1.1. Personal motivation and readiness to change

All participants reported that their motivation for abstinence was an important contributor to their success in avoiding relapse and remaining in Oxford House. Participants also indicated that their readiness to stop using led them to residential treatment and eventually Oxford House. A majority of the participants had previously attempted to stop using substances both on their own and by entering multiple treatment programs and half-way houses. The following quotes illustrate the importance of the participants’ motivation:

This time is like I needed to be here, and I really want to be here. I am not interested in using anymore. I’m tired of using drugs.

(Man from the Northeast)

Definitely, you have to want it to make it work, and this time I was ready, because it’s not the place where you go or how much money it costs, it was not that, because before I would go to programs, come out, and I would use. When I went to Oxford House I was ready to stop using and I would have done anything, gone anywhere to stop.

(Woman from the Northeast)

#### 3.1.2. Life events contributing to readiness to change

A common explanation for participants’ motivation to remain abstinent involved being tired of a variety of problems associated with substance abuse, such as being arrested, losing custody of their children, health problems, risk of overdose, homelessness, and suicide attempts. Several participants credited the legal system for providing added motivation for them to enter residential treatment and Oxford House. The following quotes further illustrate sources of participants’ motivation to remain in Oxford House and abstain from substance use:

I slept on the street, in the cold. I went for two weeks without taking a shower. I would stand on a street corner, totally disoriented, and not know where to go. I never want to go back to that.

(Man from the Midwest)

I thought about my son and I also overdosed, two people I know who used with me overdosed and died, and I went through that and thank God I am alive. So I made the decision, as I thought about my life, and not wanting to leave my son an orphan.

(Man from the Northeast)

#### 3.1.3. Residential treatment

A smaller number of participants credited residential treatment programs for their motivation to remain abstinent. These participants stated that they learned something new about addiction and paths to recovery, received support from fellow residents and treatment staff, and made connections with 12-step programs as a result of their participation in residential treatment. However, most of the participants indicated that they had participated in residential treatment many times before and as a result, their treatment experience prior to entering Oxford House did not offer them anything new, other than a hiatus from substance use and the opportunity to reflect on the consequences of their addictions.

#### 3.1.4. Twelve-step involvement

Half of the participants reported that their involvement in 12-step programs was another important contributor to their ability to remain abstinent. Other participants, however, indicated that they did not find 12-step attendance meaningful and did not relate to the focus on spirituality espoused by these programs. Additionally, the participant who had lived in an Oxford House for three years reported that he was less involved in 12-step groups than when he first entered the House, because he felt more secure in his recovery and felt less of a need for peer support. The following quotes illustrate different opinions about the role of AA and NA in recovery:

That is the foundation of it. I’m not able to do what I do for Oxford House, I’m not able to do what I do for my relationship or my job unless I’m grounded in the 12-steps. Without that there is no recovery, so that comes first, before the House, before my significant other, before my children, that is first. That’s what keeps me spiritually grounded and fed with the right information that I need.

(Man from the Midwest)

Yeah I go, but I’m not sure that I’m really into all that stuff. I’m not sure about the whole thing. It’s not for me. What helps me is to live with people who are trying to stay off drugs.

(Woman from the Northwest)

### 3.2. Therapeutic Components of Oxford House

#### 3.2.1. Mutual help

In discussions regarding the benefits of Oxford House, an overarching theme was the belief among participants that a key component of the program’s effectiveness was the absence of professionals. In other words, the importance of living with others in recovery was evident in all the interviews and participants further highlighted the power of being in charge of the program. The following quotes illustrate the value of living with recovering peers:

Staff puts too much pressure on you. It's not the same thing with someone who has been on drugs. Here we are all the same. Here we have no paperwork to complete, in the half-way houses, there is always paperwork. If you get home a little late, it's a problem. Here we have rules, but we understand each other. Here we want to change because we trust one another.

(Man from the Northeast)

We all know each others’ business and we have a common goal. The ancient Aztecs believed that when two or more people come together, they gather their individual energies and then things can happen. This is the same concept we have in Oxford House.

(Man from the Midwest)

#### 3.2.2. Sober environment

Another common theme for the majority of the participants was the role that living in a sober environment played in their recovery. Most individuals focused on the benefits of living in a substance-free home with other individuals in recovery. Others also stated that moving to a new neighborhood, away from substance-using peers was one of the most helpful elements of living in Oxford House. For others, living away from family was helpful as well. The following quotes illustrate this common theme:

I feel safe here. Here I am not exposed to the places where people are drugging and drinking. While I live here I can be sure of that, until I become stronger by working my program of recovery.

(Man from the Northeast)

Yes, my only other option was to get out on my own and I couldn’t afford that, or move in with my parents and that alone in itself is a relapse trigger. They don’t use drugs or alcohol or anything but, but just the codependency issues, enabling issues and just being over there in their house creates anxiety for some reason.

(Man from the Southwest)

#### 3.2.3. Support

Within the sober, mutual-help environment, various types of support were seen as therapeutic components of the Oxford House experience. Among these, emotional support was endorsed most often as illustrated by the following quotes:

We deal with a lot of feeling on the street where you wouldn’t be able to talk to some guy and I’m feeling like this, but it’s kind of cool that you got that here, you can talk to people.

(Man from the Midwest)

I think it helps living amongst other recovering addicts because you find yourself talking about your problems and getting advice on different issues and you are not alone. Because many times people feel like they are alone and I think that is a great tool to go along with your recovery.

(Man from the Southwest)

I think (what helps most) is the support from the other women. There is always someone to talk to, if you are feeling down someone will ask you what’s up. We talk to each other. We listen to each other.

(Woman from the Northwest)

Financial support such as the opportunity to share rent and other housing expenses and exchange information on available jobs was also mentioned by a majority of the participants, as illustrated by the following quotes:

I’ve probably experienced the best three years out of my life here. I mean all my needs are met; all I have to do is keep a job.

(Man from the Southwest)

*The jobs have been happening. My first job when I got out* (of residential treatment) *was set up through other guys in the house. You know another guy put in a letter of recommendation and I got the job.*

(Man from the Midwest)

#### 3.2.4. Accountability

Another frequently revealed theme was that residents held each other accountable, thus helping resist cravings to use drugs and alcohol. The desire to maintain the lifestyle possible as a result of living in an Oxford House (e.g., peer support, sober environment, and financial security) was mentioned by several individuals as motivation to remain sober. In this context, participants stated that they could not manipulate recovering peers the way they had manipulated significant others in the past. Therefore, they not only needed to make sure not to use drugs, but also were encouraged to focus on recovery in a broader sense. Participants also stated that the Oxford House system of chores, fines, and behavioral contracts helped support the residents’ accountability to one another. The following quotes illustrate this theme:

They will confront you and tell you, “you’re not doing anything, you’re not going to meetings, you’re not talking” and that’s how it is. They help you, do what they have to do…It’s not somewhere you can come to relax, or just to have a place to stay; it’s a program that you work.

(Man from the Midwest)

I have people watching over me and that’s a good feeling, to have people watching over me instead of having people standing over me. In the beginning, I remember, it was difficult making that adjustment. I wasn’t allowed to act up. I had to be responsible.

(Man from the Midwest)

#### 3.2.5. Less frequently-endorsed themes

Modeling, trust, respect, and freedom of choice were also identified as helpful components of Oxford House; however, these themes were endorsed by fewer participants. The following quotes from three different men from the Midwest illustrate these themes:

When people talk about their lives and I ask them questions, I pick up what I need to help me, and it is helping me.

(Modeling)

The people trust you and I am tired of people not trusting me. I have a key to the house. My opinions count, my vote counts. If I don’t raise my hand to vote during a meeting someone will ask me what I think.

(Trust and respect)

Finally someone gave me the freedom of choice, when I go to sleep, when I get up. Actually this is the first time that somebody has given me a chance to be me.

(Freedom of choice)

### 3.3. Definitions of Recovery

#### 3.3.1. Abstinence

A common theme across all the interviews was the importance of abstaining from substance use. Not only did participants see abstinence as their primary goal, but it was also presented as a requisite for remaining in Oxford House, because residents who use must enter treatment or they are evicted.

#### 3.3.2. New skills

Another common theme was that living in Oxford House allowed residents to develop new skills, such as paying bills, shopping, cooking, and keeping a job. Several residents also focused on interpersonal skills learned while living in Oxford House, such as self-disclosure and conflict resolution. The opportunity to practice interpersonal skills in the House, the need to be accountable to fellow residents, and the Oxford House system of chores and fines were given credit for changes in behavior. Quotes from three participants illustrate this theme:

In Oxford House you learn to administer your own money, pay your bills, shop, and cook. Here you learn things that you don’t know in life. The program helps us to be more responsible because we have to contribute to the house, pay the bills, and keep the house clean.

(Man from the Northeast)

You act differently; you want to talk.

(Woman from the Northeast)

I had been on vacation for 13 years and they told me “now you have to work”. It was a hard adjustment, getting used to working again and making meetings and being responsible and doing chores and all that. I remember when I first came in the House, I had a planner and I used to plan my day, I used to write everything down, get up, have breakfast, take a shower, and go to work. I had to break down my day like that. So to get up, go to work, make your paycheck stretch until the next one; those were skills that I had been informed about but I had not really put into practice.

(Man from the Midwest)

#### 3.3.3. Self-esteem/purpose

Several participants reported that as they learned new skills, they gained a sense of purpose in life, as illustrated by the following quotes from two different men from the Midwest:

I am able to make decisions and I’m able to be responsible and pay bills and on a broader spectrum I am able to be an individual part of a whole and be responsible for my individual part which contributes to the group and I do matter.

I feel useful, before I used to think that I wasn’t useful to society.

### 3.4. Culture in Oxford House

All but two of the participants interviewed reported living in Oxford Houses where they were the only Latino/a resident. The other two participants lived in a House that was composed primarily of Puerto Rican men. While the two participants living in a majority Puerto Rican House spoke about the benefits of sharing a cultural background, most of the participants who were in the minority reported that ethnic and cultural differences were not salient issues in their Houses. The following quotes illustrate the participants’ views on the role of culture in the Houses:

In this house we are all Latinos, we understand each other a little better because it’s cultural, because we Latinos have our own way of behaving, and joking, and we understand each other.

(Man from the Northeast)

I don’t see any difference at all, I mean like its one family. There are no differences I mean you’re Black, you’re White, you know, no differences at our House, I’m not sure about any other House. I haven’t heard anything from any other houses. I don’t see racism as being a problem.

(Man from the Midwest)

It (ethnicity) isn’t an issue. I get along with everyone, everybody feels like they look out for me and I feel comfortable.

(Man from the Southwest)

## 4. Discussion

Grounded theory analysis of interviews with Latino/a Oxford House members revealed their life events, readiness to change prior to coming to the Oxford Houses, and experiences of therapeutic components of this international network of mutual help recovery homes. The most frequent themes highlighted the importance of personal motivation and readiness to change, mutual help, a sober environment, social support, accountability, and the opportunity to develop new life skills. Modeling, trust, respect, freedom of choice, and participation in 12-step groups were cited less frequently as helpful aspects of the Oxford House experience.

While the study’s goal was to describe therapeutic components of Oxford House from the participants’ perspective, grounded theory methodology depends on the researchers to convey the participants’ view of reality to the reader [27]. Thus, any conclusions drawn from this study are inherently limited by the impact of the researchers’ personal experiences and biases [27]. However, having two independent coders and discussing findings with other researchers and with the study’s participants enhances the credibility of the study’s findings [25]. Additionally, the nature of the grounded theory methodology employed in the current study does not allow for broad generalization of the findings beyond the current sample [28]. However, the themes that emerged from the current analysis are supported by empirical literature on substance abuse treatment and mutual help groups.

Recent research indicates that personal experiences of loss and trauma along with other substance-related problems encourage individuals to seek treatment and may provide motivation for maintaining abstinence [30,31]. Additionally, numerous empirical studies support the importance of motivation to stop using as a predictor of length of stay in various types of substance abuse treatment programs and 12-step groups [32,33]. Legal involvement prior to entering treatment predicts longer stays in various types of programs and does not have a negative impact on treatment satisfaction or outcome [34,35]. The current study’s findings suggest that personal motivation to stop using and legal involvement may facilitate participation in mutual help interventions.

In the current study, mutual help emerged as a major therapeutic aspect of participation in Oxford House. Throughout their interviews, participants consistently indicated that Oxford House was helpful to them because they developed relationships with peers based on common experiences and goals, mutual understanding, trust and respect. The inability to manipulate other House residents who held them accountable for their actions, was also frequently cited as an important component of the Oxford House experience. This finding is consistent with literature indicating that participation in mutual help interventions such as 12-step groups is one of the most powerful predictors of long-term abstinence among individuals in recovery [3–5].

Oxford House [14] documents indicate that providing sober housing and support for individuals in recovery are two of the organization’s central goals. In the current study, the importance of living in a sober home away from relapse triggers was a recurrent theme. Additionally, having access to emotional and, secondarily, financial support were also seen by participants as therapeutic components of the Oxford House experience. Researchers have only recently begun to explore the impact of environmental variables on long-term abstinence, and this early research indicates that factors such as the availability of illicit drugs in one’s neighborhood have an impact on rates of substance use and recovery [36,37]. Research also points to the key role of social support in recovery [1,16]. Furthermore, existing evidence indicates that abstinence-specific, as opposed to general support, is a more powerful predictor of substance use outcomes [38]. However, in the current study, participants were more likely to focus on the value of being able to share feelings, talk about experiences, and seek advice from peers, while the value of abstinence-specific support was mentioned less often.

Another commonly-endorsed theme related to the therapeutic value of being held accountable by other recovering individuals. Participants indicated that the desire to remain abstinent, financially stable and involved in relationships with Oxford House peers helped them resist cravings and refrain from substance use. Participants also talked about the ways in which peer support and enforcement of House policies and procedures helped them to avoid relapse triggers. Accountability in mutual help groups in general and Oxford House in particular has not been widely studied. However, the salience of this aspect of the Oxford House program is consistent with the therapeutic community model of substance abuse treatment, particularly its reliance on the use of the peer community and the programs’ norms and structure to facilitate change [39]. Participants’ emphasis on the therapeutic impact of the various contingencies provided by the Oxford House program is also supported by studies that support the effectiveness of contingency-management interventions for individuals in recovery [40,41].

Finally, participants described their recovery in broad terms, focusing on the various skills and abilities they were learning as a result of living in an Oxford House. These included basic life skills such as cooking and managing a budget as well as various interpersonal abilities. Several participants also spoke about gaining a greater sense of self-esteem while living in Oxford House. These findings are consistent with a broader conception of recovery beyond mere abstinence [42] and point to a need to devote greater attention to skill development as an outcome of substance abuse interventions.

As previously stated, only half of the study’s participants indicated that participation in 12-step groups was helpful to their recovery. Most of the other individuals reported that they could not relate to the groups’ emphasis on spirituality. These findings are also consistent with literature indicating that many individuals in recovery do not attend 12-step groups [11,12,43]. The outcomes of individuals who do not find 12-step programs useful and their sources of support in their recovery merit further attention from researchers.

The majority of the study’s participants reported that they did not experience problems in the Houses as a result of cultural differences (see also [24]). However, most of the individuals interviewed were U.S.-born, English-speakers who reported many experiences living in racially-mixed settings (e.g., prisons, college dormitories, residential treatment programs, and half-way houses). Thus, any attempts to generalize the study’s findings to other Latinos/as seeking substance abuse recovery are limited by the size and homogeneity of the current sample. Participation in mutual help groups may be influenced by cultural variables such as language preference and proficiency, comfort interacting with individuals from other ethnic groups, as well as cultural values and practices [10–12].

In addition to the cultural factors just mentioned, participants were mostly men in their ‘30s who had long histories of poly-substance use, numerous prior substance abuse treatments, and involvement in the criminal justice system. It is likely that the individuals who participated in the current study are representative of Latino/a Oxford House residents, but not of Latinos/as in need of substance abuse interventions.

Another limitation is that the sample for the current study is small. However, there was general agreement among participants on all the major categories of the therapeutic components of Oxford House (i.e., readiness to change, mutual help, sober environment, support, accountability, and recovery). Generally, samples for grounded theory studies tend to be rather small to allow for the type of in depth data analysis required [25,28]. Additionally, qualitative researchers consider a sample to be sufficiently large when participants repeatedly express agreement on the main themes that emerge from data analysis and major inconsistencies in participants’ responses are not evident, as was the case in the current study [25,28]. Furthermore, differences in participant responses based on gender, national origin, region of the country, and telephone versus face to face interviews were not apparent in the data analysis.

A further limitation may involve reliance on interview methods as opposed to using multiple methods of data collection [25,29]. In addition, a series of interviews may have provided richer data on the Oxford House experience and more nuanced aspects of the participants’ cultural affiliation. Multiple interviews may have also enhanced rapport between researcher and participants, possibly leading to more valid data [44]. However, the researchers were familiar with Oxford House and had previously used both qualitative (i.e., interviewing, observation, review of documents) and quantitative methods to study this organization [15–18]. Furthermore, the interviewer’s familiarity with Oxford House and with the experiences of Latinos and Latinas appeared to enhance rapport and facilitate disclosure among participants.

We have found several similarities in the model (grounded theory) found here and what has been found for the majority of people in these kinds of treatment settings in general. Most of the findings are not dissimilar to what has been reported by others about these kinds of treatment settings. The current findings indicate that at least some Latinos/as find mutual help communal programs such as Oxford House to be a helpful component of their recovery. It will be necessary to further investigate the experiences of more diverse groups of Latinos/as in mutual help communal programs using quantitative and qualitative methodologies. Studies with larger more representative samples comprised of individuals differing in terms of language preference, place of birth, generation in the U.S. and other indicators of cultural affiliation are needed. It is possible that Latinos/as who are more strongly affiliated with their ethnic cultures may not find the Oxford House program as helpful as the current study’s participants. A culturally-modified intervention may be more appropriate for these individuals.

Although the focus of this study was on Latinos and Latinas, and their specific experiences, the findings are also of importance as larger proportions of minorities than Whites are mandated to treatment by the criminal justice system [45], and African Americans, when they do receive treatment, are more likely to enter care through legal and court channels [46]. Additionally, the current findings point to potentially therapeutic components of mutual help residential recovery interventions for Latinos and Latinas; these findings also await validation with larger samples with different ethnic groups. Research indicates that mutual help communal setting such as Oxford House may enhance substance abuse recovery [18]. These settings likely contribute to increased motivation and facilitate various types of therapeutic experiences which need to be studied further. The therapeutic impact of mutual help, social support, accountability, a sober environment and the opportunity to develop life skills in a communal setting needs to be established.

## Appendix A: Interview Guide

This interview is part of the DePaul Study of Oxford Houses. The purpose of the DePaul study is to learn about the experiences of Oxford House members and to understand how Oxford House helps men and women recover from alcohol and drug addiction. We also hope that this information will be helpful to Oxford House, Inc, to the various state chapters, to people who run treatment programs, and to those who fund substance abuse services. People who are working on their recovery are the best sources of information on what helps. The reason why I am talking to you tonight is to learn more about how Hispanic/Latino men and women experience living in an Oxford House.

I am going to be taping this interview to make sure that I remember what you tell me. I will destroy these tapes once I transcribe them. I would like to review some of the things that we said in the consent form and want to remind you that what we discuss is confidential. Remember, you may stop the interview at any time. If you have any questions about anything I ask, or if something is not clear, please ask and I will try to clarify things. You don’t have to answer questions that make you feel uncomfortable. If you prefer not to answer a question, please let me know. If anything we discuss stirs up difficult feelings, please let me know. Do you have any questions at this time? Are you ready to start?

1. We find that people come to Oxford House in many ways and from different places. I’d like to learn how you came to live in the (house name) Oxford House*? (Who referred you? what did they tell you about it? how did you decide to move in?)*
2. Do you remember your thoughts and feelings as you decided to join the house? *(Any apprehensions or concerns?)*
3. Tell me about your family’s reaction to your decision to live in Oxford House? (*Probe for parents, siblings and other family members. Probe for verbal and non-verbal ways family members expressed feelings about the move*).
4. Tell me about what it’s been like for you to live in Oxford House so far? (*Probe for what person finds helpful/unhelpful about OH experience. Ask for specific stories to illustrate their experiences*)
5. It seems that you identify with being Latino (Puerto Rican/Mexican, etc.) (or, you talked about some of your concerns before coming live in the house). What is it like for you to live in a house were there are no other Latinos? *(Probe for any differences that might come up due to culture and how these are addressed)*
6. What do you think are some of the reasons why Latinos may or may not be coming to Oxford House?
7. What suggestions do you have for attracting more Latinos to Oxford House? (*What changes do you think need to take place to attract more Latinos to OH?*)
8. Is there anything about your Oxford House experience we have not discussed that you think might be important for us to understand?
